# Cardiac Optogenetics in Atrial Fibrillation: Current Challenges and Future Opportunities

**DOI:** 10.1155/2020/8814092

**Published:** 2020-10-27

**Authors:** Mariana Floria, Smaranda Radu, Evelina Maria Gosav, Aurelian Corneliu Moraru, Teodor Serban, Alexandru Carauleanu, Claudia Florida Costea, Anca Ouatu, Manuela Ciocoiu, Daniela Maria Tanase

**Affiliations:** ^1^Emergency Military Clinical Hospital, 7-9 General Henri Mathias Berthelot Street, 700483 Iasi, Romania; ^2^“Grigore T. Popa” University of Medicine and Pharmacy, 16 University Street, 700115 Iasi, Romania; ^3^Cardiology Clinic, “Prof. Dr. George I.M. Georgescu” Institute of Cardiovascular Diseases, Iasi 700503, Romania; ^4^Romanian Academy, Romania; ^5^Department of Obstetrics and Gynecology, “Grigore T. Popa” University of Medicine and Pharmacy, 700111 Iasi, Romania; ^6^Department of Ophthalmology, “Grigore T. Popa” University of Medicine and Pharmacy, Iasi 700115, Romania; ^7^2nd Ophthalmology Clinic, “Prof. Dr. Nicolae Oblu” Emergency Clinical Hospital, Iași, Romania; ^8^IIIrd Medical Clinic, “Sf. Spiridon” Emergency Hospital, 1 Independentei Street, 700111 Iasi, Romania; ^9^Pathophysiology Department, Grigore T. Popa University of Medicine and Pharmacy, 16 University Street, 700115 Iasi, Romania

## Abstract

Although rarely life-threatening on short term, atrial fibrillation leads to increased mortality and decreased quality of life through its complications, including heart failure and stroke. Recent studies highlight the benefits of maintaining sinus rhythm. However, pharmacological long-term rhythm control strategies may be shadowed by associated proarrhythmic effects. At the same time, electrical cardioversion is limited to hospitals, while catheter ablation therapy, although effective, is invasive and is dedicated to specific patients, usually with low amounts of atrial fibrosis (preferably Utah I-II). Cardiac optogenetics allows influencing the heart's electrical activity by applying specific wavelength light pulses to previously engineered cardiomyocytes into expressing microbial derived light-sensitive proteins called opsins. The resulting ion influx may give rise to either hyperpolarizing or depolarizing currents, thus offering a therapeutic potential in cardiac electrophysiology, including pacing, resynchronization, and arrhythmia termination. Optogenetic atrial fibrillation cardioversion might be achieved by inducing a conduction block or filling of the excitable gap. The authors agree that transmural opsin expression and appropriate illumination with an exposure time longer than the arrhythmia cycle length are necessary to achieve successful arrhythmia termination. However, the efficiency and safety of biological cardioversion in humans remain to be seen, as well as side effects such as immune reactions and loss of opsin expression. The possibility of delivering pain-free shocks with out-of-hospital biological cardioversion is tempting; however, there are several issues that need to be addressed first: applicability and safety in humans, long-term behaviour, anticoagulation requirements, and fibrosis interactions.

## 1. Introduction

Cardiac optogenetics is a novel research field, involving delivery of microbial light-sensitive proteins called *opsins* to excitable heart cells, thus enabling either light-based depolarization or hyperpolarization. Originally used in neurology, recent studies have shown that optogenetics can be used to terminate arrhythmias, pace, or even resynchronize hearts. In pacing, optogenetics would have the much-desired benefit of providing a more physiological and synchronized contraction, as opposed to classical right ventricular pacing. Carefully programming opsins and light pulses would even allow cardiac resynchronization. This review focuses on the potential applications of cardiac optogenetic actuators in atrial fibrillation (AF) rhythm control therapy. We will discuss the general principles of cardiac optogenetics, their use in cardiology (pacing, resynchronization, and biological cardioversion/defibrillation), and possible advantages and limitations of these techniques in the treatment of AF.

## 2. Cardiac Optogenetics

### 2.1. Definition

The term “optogenetic” refers to the delivery of light-sensitive proteins to cardiac excitable cells, rendering them responsive to certain wavelength light pulses [1–6]. There are mainly two types of opsins used—sensors and actuators. Sensors seem promising in monitorization and even *arrhythmic substrate characterization* as they emit light themselves in response to several changes in the intracellular milieu (i.e., changes in calcium concentration/disposition or in intracellular pH) [7–10]. In contrast, actuators *alter* the membrane potential in response to light, leading to either depolarization or hyperpolarization. This review will focus on the latter and their possible uses in arrhythmia management, especially in atrial fibrillation.

### 2.2. Preferred Opsins

Optogenetic actuators form transmembrane domains which are covalently bound to a light-sensitive cofactor, retinal. Upon applying light pulses, light photons determine a change in the configuration of retinal from all-trans-retinal (ATR) to 13-cis-retinal, thus enabling ion influx [2]. The three main classes of opsin actuators in cardiac optogenetics are bacteriorhodopsins, halorhodopsins, and channelrhodopsins (Table 1).

Bacteriorhodopsins and halorhodopsins are pumps with their properties limited to exchanging 1 photon per each chloride and proton, respectively. Thus, the resulted electrical current will be of a lower amplitude as compared to the one resulted from the activity of channelrhodopsins. However, it has the advantage of being less influenced by the changes in the transmembrane potential [2].

The channelrhodopsins are nonselective cation channels, allowing natrium and calcium influx upon 480 nm blue light stimulation [6–10]. The resulting fast membrane *depolarization* is just as rapidly reversible in the absence of light [10–13]. Bruegmann et al. achieved optical pacing by fusing ChR2 into embryonic mouse stem cells, which developed ventricular arrhythmias after long light pulse therapy due to an increase in resting membrane potential [13]. Consequent computational modeling studies revealed that a 10 ms, 0.5 mW/mm^2^ blue light pulse is enough to determine action potentials in human atrial, ventricular, and Purkinje cells [11], while it is known that the latter require the lowest energies for optogenetic pacing, followed by atrial cardiomyocytes [5].

Of course, attempts have been made to improve the efficiency of the various channelrhodopsins. As such, several hybrids were developed, out of which ChR2 with H134R mutation shows the most promise, as this mutation triples the channel conductance and lowers the associated kinetic loss [14–19].

The fact that red light has better tissue penetration prompted the development of the red light-shifted ChR2 (ReaCh), with its alternatives ReaChR, ChRimson, and ChRonos. They possess decreased kinetics and slower opening in response to short light pulses [20]. This kind of hybrids with decreased kinetics is of particular interest in Short-QT syndrome, where the heterogenicity of action potential duration is the cause of reentrant and autonomous atrial and ventricular arrhythmias. Studies on atrial cardiomyocytes expressing ChR2 showed action potential (AP) prolongation upon light stimulation [21].

There have also been attempts of optogenetic cardiomyocyte inhibition, mostly by using hyperpolarizing opsins in order to inhibit cardiac action potential [22, 23]. Two of the mostly used hyperpolarizing opsins are NpHR and ArchT that have the potential of forcing repolarization on the atrial myocytes, virtually eliminating any depolarization in the spontaneously depolarizing cells. This is of particular interest when used in abnormal myocardium in which cells struggle to maintain a normal diastolic membrane potential. Moreover, ArchT was able to isolate the electrical activity of one cell culture from another, a feature that may prove useful during certain ablation procedures such as pulmonary vein isolation [24–27].

As the majority of the hyperpolarizing opsins are pumps (most frequently used the bacteriorhodopsins-Arch) and as stated above, the resulting electrical current intensity is of a small amplitude as it is based on 1 : 1 ion exchange.

Recently, Kopton et al. have attempted to determine cardiomyocyte inhibition through a chloride channelrhodopsin [22]. GtACR1 is a channel that allows upon green light stimulation chloride influx with minimal cationic influx. Interestingly, although it is a chloride-based opsin, it leads to membrane depolarization while maintaining a negative resting membrane potential. By forcing cardiomyocytes into the depolarizing state, it prevents another depolarization [22]. When compared to Arch3, it has been shown to require three times lower light intensities to produce an electrical current of the same intensity. Moreover, studies have even shown a better efficiency in inhibiting cardiomyocyte AP as compared to Arch3 and that the chloride influx can be controlled by adjusting light pulse intensity and duration.

Introducing simultaneously two different channelrhodopsins in the human cardiomyocytes is an interesting concept that could lead to a complete electrical control of the heart. The problems lie though in the different distributions of these channels throughout the cardiac tissue [27–31]. For example, some myocytes may express both channels; some may have just one and some none. This could give birth to myocyte action potential heterogeneity, leading to a paradoxical and undesirable proarrhythmic effect. This may raise an issue especially in structurally abnormal hearts that already have a heterogeneous action potential. Moreover, these patients exhibit increased fibrosis levels, which may also interfere with opsin expression and function while being arrhythmogenic on its own.

Hence, more and more studies have shifted towards searching for an opsin capable of both depolarization and hyperpolarization. Pluripotent stem cell-derived cardiomyocytes were coupled with ChR2 (used for its depolarizing properties in blue light) and halorhodopsin (NpHR1.0) (hyperpolarizing under yellow light) [32–37]. Stimulating with 0.5 to 1.5 Hz blue light gave rise to depolarizing electric potentials that could be later terminated under direct yellow light [31]. A different approach consisted of modifying fibroblasts into expressing both ChR2 and ArchT, leading then to the ability of both pace and inhibited cardiomyocyte action potential [38].

### 2.3. Opsin Delivery

If choosing an appropriate opsin is the first step in implementing optogenetics, choosing a proper delivery method is just as important. So far, three methods have been attempted, each with its own advantages and disadvantages and varying from gene delivery to opsin delivery per se: using transgenic specimens, direct cellular delivery, and gene delivery via viral vectors [39, 40].

#### 2.3.1. Transgenic Animals

The first studies regarding optogenetics involved transgenic zebra fish and mice. Arrenberg et al. have induced cardiac arrhythmias and conductive disorders by delivering light in zebra fish expressing both ChR2 and NpHR [30]. Similar findings have resulted from studies on transgenic *Drosophila melanogaster* [41] and transgenic mice [13]. However promising these findings are, their use is limited in the human heart, motivating the development of newer gene delivery techniques.

#### 2.3.2. Direct Cellular Delivery

Direct opsin delivery can be achieved either through intracoronary injections or direct myocardial delivery of opsin-expressing donor cells. So far, in cardiac optogenetics, the second method was the preferred one and one of the first attempts involved delivering human embryonic kidney (HEK) cells expressing ChR2 to rodents' ventricular myocardium [5]. Once delivered, the ChR2-expressing HEK cells attached to the surrounding cardiomyocytes, forming *tandem cell units* (TCU) that contracted together upon blue light stimulation [5]. Nussinovitch et al. modified fibroblasts into expressing ChR2 and then cocultured them with either human stem cell cardiomyocytes or neonatal rodent cardiomyocytes [38]. Upon blue light stimulation, the ChR2-expressing fibroblast initiated the culture depolarization and contraction. The same authors then managed to simultaneously express a hyperpolarizing and depolarizing opsin in the cardiomyocyte culture.

There are several advantages of using a direct opsin delivery method. As opposed to intravenous systemic delivery, it requires smaller viral doses and the risks of triggering an immune response are lower. In addition, the genetic engineering per say takes part outside of the body, making it more controllable and more easily accepted from an ethical point of view. One problem that arises when using the TCU method is that of the long activation times when using a single TCU, activation that could be even more prolonged in cases of altered cardiomyocytes in a heart failure patient. This could be tackled by using more TCU coupled to the same tissue. Other disadvantages include the risk of patchy distribution, which would render attempts of optogenetic defibrillation inefficient. Not only this but also the survival of the transplanted cells beyond several months has not been proven [38]. Of note, direct cellular delivery has not been achieved in whole hearts. Moreover, this method was not applied in a whole heart.

#### 2.3.3. Systemic Gene Delivery

By far, the most widely used method of delivery is the intravenous systemic delivery [2]. It has the advantages of homogenous myocardial expression, but at the price of requiring higher opsins and viral vector concentration [2].  Table 2 summarizes the advantages between systemic and direct intramyocardial opsin delivery.

In order to achieve effective systemic opsin delivery, the use of cardiac specific vectors and/or promoters is required. Adenoviruses (AAV) are the preferred viral vectors used [39]. There are twelve existing viral serotypes (AAV1–AAV12) with various tropisms. To achieve cardiac opsin delivery, the choice is limited to a cardiac-specific serotype, such as AAV9 (the highest cardiac tropism) [39]. On the other hand, cardiac specificity alone is not enough to characterize a good delivery method. Long-term expression is also necessary when it comes to finding a long-term solution for arrhythmia treatment. A study favored the use of AAV1 serotype in heart failure cardiomyocytes because it assured long-term expression in spite of a more moderate specificity [40]. Another study proved that AAV1 and AAV6 expression has preferential efficiency in vitro, while AAV9 may be more efficient when used in vivo [42–44].

Several authors show that 90% ChR2 expression can be achieved with only one AAV vector B-actin promoter delivery [45, 46]. Reports show proper 1-year opsin expression in rodents after using this method of delivery [46].

AAV delivery is associated with the fewest side effects in comparison with the other techniques; however, its main limitation lies in its small carrying capacity (4.7 kb single-stranded DNA genome), potentially limiting the complexity and number of the channels that can be coded [47–51]. The main concern regarding AAV vectors remains the associated immunogenicity that would render some patients poor candidates to gene delivery therapy [48]. For the above-mentioned reasons, some authors consider viral delivery unsafe for in vivo use [50].

Regarding other viral vectors, there have been attempts of using lentiviruses as a delivery method, but several disadvantages precluded wide use. In a recent study, rodent ventricular cardiomyocytes expressing lentivirus-delivered ChR2 underwent certain morphological changes with increased heart rate [52].

### 2.4. Light Systems

Delivering appropriate illumination is crucial to obtaining the desired optogenetic effect. There are several difficulties in developing illumination systems in optogenetics [51–64]. The low tissue penetration of visible light is one of them, especially in patients requiring defibrillation. Developing a functional in vivo illumination system is another problem, as its design should take into consideration providing constant specific wavelength illumination intensity in a beating heart. Furthermore, cardiac mechanics could change over time, for example, with HF progression, left bundle branch development, hypertrophy, or even certain arrhythmias (atrial fibrillation) [60, 61].

So far, researchers have created *μ*-ILEDs with various characteristics that have been proven efficient in vitro and in vivo. The need to have multisite illumination required in defibrillation was addressed by Xu et al. who created an integumentary *μ*-ILED membrane [61].

Regarding the optimal illumination intensity of such *μ*-ILEDs, the authors reported a sufficient 5 mW to efficiently illuminate 0.024 mm^2^ and stimulate ChR2 [62]. Researchers have extrapolated and found that 1-second stimulation of 208.3 mJ suffices for determining efficient optogenetic stimulation in 1 mm^2^ [64]. Moreover, it seems that delivering 300 ms light pulses using 960 *μ*-ILEDs would require a similar amount of energy as an internal defibrillator-delivered shock 15 J [64, 65]. Interestingly, Boyle et al. affirmed that such a setting would allow atrial fibrillation cardioversion [66].

A different illumination solution came from Huang et al. who proposed using nanoparticles that could convert to specific wavelength light upon stimulation with a form of deep penetration energy, such as X-rays or magnetic field [67].

An efficient illumination system that would be functional in humans remains to be developed; however, it must be noted that all implantable systems will have the risk of infections.

### 2.5. Electrophysiological Monitorization

To provide optical stimulation, apart from a light source, an optical sensor is also required.

Optical sensors can be a calcium indicator protein (GCaMP), a voltage-sensitive fluorescent protein (VSFP), or a red calcium indicator protein and are usually formed by attaching a sensing domain to one or more fluorescent proteins. The sensing domain will change its kinetic properties when interacting with light [50–54]. Some of the first voltage sensors used were the styryl dyes Di-4-ANEPPS, Di-8-ANEPPS, and RH-237. However, their efficiency has been proved limited creating overlap waves and tissue scattering after blue-green light delivery. Di-4-ANBDQPQ is a newly discovered near-infrared dye, with an absorption peak higher than that of hemoglobin that has been used in projecting the light in various forms in order to modify and control the depolarization pattern of ventricular myocytes with potential implication in ventricular arrhythmia therapies [55]. On the other hand, calcium dyes (Fura-2, Rhod-4, and Fluo-4) give stronger signals but their use is limited by their transient emission prolonging effects and ability to obliterate cell kinetics [56, 57].

An alternative is represented by genetically modified calmodulin-sensing calcium indicators (GCaMP variant and GECISs). This kind of sensors has been used with success in the recording and better characterization of the myocardial calcium gradients and metabolism in rats [58]. Forster resonance energy transfer (FRET) sensors including Twitch and TN-XL have lower amplitude responses compared to GCaMPs, but they contain more fluorescent proteins that give them radiometric properties [53]. The hybrid sensor GCaMP-GR is currently under research, with improved functions [59].

## 3. Cardiac Optogenetics in Atrial Fibrillation

Cardiac arrhythmias, either supraventricular or ventricular in origin, may become life-threatening by reducing cardiac output and leading to hemodynamical instability [64–70]. Ventricular tachyarrhythmias such as ventricular tachycardia and ventricular fibrillation are most frequently associated with increased mortality and sudden cardiac death [70]. On the other side of the spectrum, AF is not immediately life-threatening (unless associated with a high ventricular rate or with an extensive acute thromboembolic event). However, in time, AF patients also present with increased mortality, decreased quality of life, and increased risk of heart failure development and progression [71]. Several authors agree on the benefits of restoring and maintaining sinus rhythm, especially in heart failure patients [71–74]. Doing so early in AF progression is even more beneficial, since sustained AF is associated with a degree of left atrium structural and functional remodelling which, in time, will render future sinus rhythm restoration strategies inefficient [73].

So far, restoring sinus rhythm in AF patients can be achieved through either pharmacological strategies, electrical cardioversion, or ablation therapies (catheter or surgical ablation). Treatment with either class IC or class III antiarrhythmics may be efficient; however, long-term administration might be associated with side effects such as thyroid disease (amiodarone) or proarrhythmic effects (especially class IC). Moreover, the latter should be avoided in structurally abnormal hearts [71]. In certain conditions (either hemodynamically unstable patients, either elective after three weeks of proper anticoagulation), electrical cardioversion may be an option; however, it requires sedation, and it may be painful nonetheless and therefore remains limited to hospitals only.

Implantable atrioverters have been developed in the past but were poorly tolerated by patients due to frequent shocks and associated pain [75, 76]. So far, ablation therapies remain the mainstay for sinus rhythm restoration [71]. Cardiac optogenetics may provide a novel solution to AF cardioversion and arrhythmia defibrillation, providing pain-free shocks that would restore sinus rhythm.

### 3.1. Mechanisms of Defibrillation and Cardioversion

There are several mechanisms explaining arrhythmia initiation and maintenance, including a trigger, a reentry circuit (either functional or anatomical), or a rotor. One of the most common mechanisms involves arrhythmia initiation through an ectopic diastolic trigger, leading to subsequent calcium influx. The resulting depolarization front may give rise to reentry when encountering a conduction block given by a nonexcitable area. The latter may be either a scar (i.e., postmyocardial infarction), leading to anatomical reentry, or an area with different conduction/electrophysiological properties, leading to functional reentry. Of note, the reentry in itself is dynamic, able to switch between functional and anatomical in the same patients during the same arrhythmia [70].

In addition, inhomogeneous refractory periods may give rise to rotors. They are practically spiral-like disorganized electrical activity gravitating around an unexcitable core [77]. They have been identified in AF, and recent ablation strategies target rotor ablation [78]. While the presence of a single reentry circuit/rotor leads to monomorphic tachyarrhythmias, additional conduction blocks/myocardial scars/several rotors determine polymorphic arrhythmias such as AF, polymorphic ventricular tachycardia, or ventricular fibrillation [70].

Through cardioversion/defibrillation an R-wave synchronized/random electrical shock is delivered to the heart, in the attempt to terminate the ongoing arrhythmia. Although the exact mechanisms of cardioversion and defibrillation are not fully understood, they most likely restore sinus rhythm and prevent arrhythmic depolarization front propagation through filling of the excitable gap (the excitable myocardium between the last arrhythmic front and the beginning of the next) [70, 79]. The excitable gap can also be reduced by increasing the cardiac wavelength, which is in turn defined by the product of cardiac conduction velocity and AP duration. By prolonging the latter, optogenetic defibrillation will lead to filling of the excitable gap [23].

Upon cardioversion/defibrillation, the delivered electric shock determines massive depolarization with subsequent AP prolongation, thus rendering the depolarized myocardium refractory and unresponsive to the next arrhythmic front through AP prolongation. The resulting depolarizing front collides with the arrhythmic front and inhibits each other.

There are several disadvantages to electrical defibrillation/cardioversion. First, the induced depolarization is transient (ms). Secondly, it is painful and requires sedation, which precludes, in the case of cardioversion, out-of-hospital use. Thirdly, the defibrillation wave has an inhomogeneous effect on the cardiomyocyte membrane potential, also increasing the number of hyperpolarizing cells. At this level, through an electrical gradient between hyperpolarized and depolarized cells, arrhythmic reinitiation can occur and it is one of the causes of defibrillation failure. This could be prevented by ensuring homogenous Na^+^ channel recovery, which would allow proper conduction and timing of the depolarizing front to collide with the arrhythmic one [79]. It is here where cardiac optogenetics could benefit patients, by ensuring continuous, pain-free depolarization, without the destruction of either myocardium or surrounding structures. Moreover, the ability to induce cardiomyocytes into expressing both depolarizing and hyperpolarizing opsins means a higher degree of myocardial AP control.

### 3.2. Mechanisms of Optogenetic Cardioversion in AF

Cardiac optogenetics may use both depolarizing and hyperpolarizing opsins in order to terminate arrhythmias. When using depolarizing opsins, such as ChR2 or CatRh, defibrillation/cardioversion may be achieved either through filling of the excitable gap or through induction of a transmural conduction block [70].

#### 3.2.1. Depolarizing Opsins: Conduction Block

Stimulating ChR2-expressing cardiomyocytes induces depolarization through Na^+^ inflow, leading to AP prolongation and increase in refractory period. This depolarization is continuous, as opposed to the transient electrical shock-delivered depolarization. This mechanism is efficient in arrhythmia termination if its underlying mechanism is dependent on conduction in the illuminated area [2]. Moreover, opsin-based depolarization could diminish the conduction speed gradient between different regions, which in itself is arrhythmogenic.

The disadvantage of this method is that, in contrast to optogenetic pacing, optogenetic defibrillation requires transmural illumination for successful arrhythmia termination. Studies focusing on ventricular tachycardia termination confirmed the necessity of transmural homogenous ChR2 expression and illumination delivery of constant intensity throughout the surface to achieve successful arrhythmia termination [70, 78–82].

Transmural opsin expression and appropriate illumination raise several challenges. First, blue light (470 nm) has a relatively weak tissue penetration; thus, classical ChR2 may be insufficient. The solution was the use of modified ChR2-ReaChR2 with a so-called *red light shift*, making it responsive to 670 nm red light [70, 80]. On the other hand, the thinner atrial myocardium might enhance conduction block achievement. Bruegman et al. have successfully terminated AF in connexin 40-mutated mice. The authors induced AF in susceptible mice by shortening refractory period using diazoxide and showed that both epicardial illumination successfully reduced AF in ChR2-expressing mice [81]. They proposed both filling of the excitable gap and conduction block as possible mechanisms explaining AF optogenetic cardioversion, differing with the intensities of the light pulses. As such, lower intensity light pulses most likely could terminate AF through filling of the excitable gap while higher intensity light pulses terminate AF by inducting conduction block. However, they highlight that filling of the excitable gap is less likely to lead to successful AF optogenetic cardioversion, since the excitable gap is small, inhomogeneous, and hard to identify; therefore, random optogenetic-based stimulation at this level would be inefficient. Interestingly, CD45+ lymphocyte infiltrates were reported in ChR2 mice; however, there was not a sustained immune response against both the opsin and the vector (AAV2/9). Moreover, there was substantial 6-month postdelivery atrial ChR2 expression.

Another example of optogenetic conduction block in arrhythmia termination is the study conducted by Feola et al. in which the authors attempted rotor-guided optogenetic (CatCh) atrial arrhythmia ablation in cardiomyocyte monolayers [83]. Only a line of conduction block through the rotor core and spanning to at least one unexcitable edge was able to terminate arrhythmic activity, while conduction block around the core was inefficient. On the other hand, applying a linear conduction block outside the rotor core was also unsuccessful, despite reaching the opposing two unexcitable regions. The authors highlight however that using atrial cardiomyocyte monolayers led to the induction of stable rotors, which may be very different from real-life settings of fibrotic and structurally remodeled hearts with subsequent unstable rotors and inhomogeneous electrical conductions [84].

**Figure 1:**
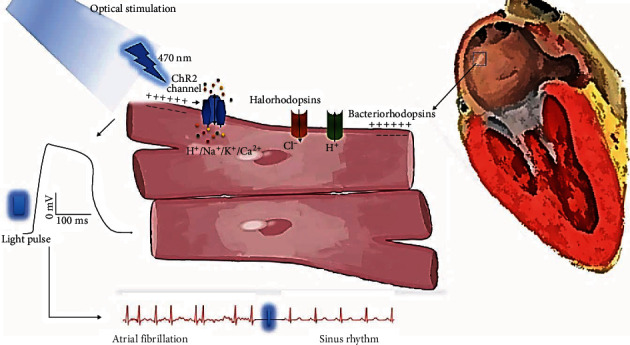
Mechanisms of optogenetic cardioversion in atrial fibrillation. After gene delivery, channelrhodopsin 2 (ChR2) from opsin-expressing cardiomyocytes stimulated by diode/laser light produce inward photocurrents of nonselective cations and evoke cell depolarization (electrical response); bacteriorhodopsin (BR) and chloride pumps like halorhodopsin (HR) have inhibitory/hyperpolarizing effects.

In contrast, Rappel et al. show that ablating near the core might destabilize the rotor in structurally altered hearts [85]. Recently, Nyns et al. delivered through right atrial gene painting the red light-shifted ChR2 (ReaCh) using AAV2/9 [35]. Four weeks after gene delivery, there was right atrial transmural ReaCh expression with minimal extraright atrial expression (6% in left atrium, 0.3% right ventricle, and 0.1% left ventricle) and no reported ventricular arrhythmias. Induced AF was successfully terminated with a single epicardial 470 nm, 3.5 mW/mm^2^, 20 mm^2^ light pulse. Narrowing the surface to 10 mm^2^ while maintaining the initial light intensity and wavelength still resulted in a 95% AF termination rate. However, the success rate dropped to 65% and 35% when the surface was narrowed to 5 and 2.5 mm^2^, respectively. They remarked that successful arrhythmia termination was dependent on using a light pulse duration longer than the AF cycle. Interestingly, the authors reported no spontaneous AF termination and no termination when both out-of-spectrum and out-of-arrhythmic-event light pulses were applied. Furthermore, atrial flutter episodes were also successfully terminated using the same technical specifications. The authors further developed a cardiac optogenetic-based closed-chest hybrid system, capable of both detecting and terminating AF. The 100% AF detection accuracy was followed after a 10 s delay (to ensure that the arrhythmia is sustained) by a 470 nm, 500 ms, 2.5 mW/mm^2^ light pulse, resulting in a 96% AF termination rate. Regarding possible complications/limitations, the authors reported no local hyperthermia with a maximum of 36.8°C preillumination and 37.2°C postillumination. The mechanisms involved in optogenetic cardioversion of AF are presented in Figure 1.

Interestingly, the authors raise the possible optogenetic difficulties in the presence of myocardial fibrosis, frequent in AF patients. In an attempt to evaluate the applicability of cardiac optogenetics in fibrotic hearts, Boyle et al. conducted computer simulations based on fibrotic patients' hearts late-gadolinium enhancement-cardiac magnetic resonance scans, but using atrial tachycardia models. The authors took into consideration the atrial fibrotic burden and analyzed whether gene-delivered ChR2 blue light stimulation would terminate atrial tachycardia. Comparing general (endocardial) and localized illumination, they proved that by delivering light pulses targeting the specific arrhythmic isthmus of a duration longer than atrial tachycardia cycle (1000 ms) resulted in 94% termination rate. When lowering the light pulse duration to 100 ms, the success rate dropped to 54% [86]. The authors also highlight that transmural atrial illumination might be easier to achieve given the thinner walls and that failure to terminate atrial tachycardia was due to incomplete transmural illumination. Another interesting finding is that in their study using ChR2 and subsequently blue light pulses was sufficient for arrhythmia stimulation, with no need of switching to ReaCh. Bingen et al. also showed that AF may be terminated using 500 ms, 38 *μ*W/mm^2^ blue light pulses on CatCh-expressing rodent atria myocytes [87].

#### 3.2.2. Depolarizing Opsins: Filling of the Excitable Gap

The mechanisms are similar to antitachycardic pacing; however, it requires the exact knowledge of the location of the excitable gap, thus a precise optical mapping [82]. One advantage is the requirement of short and repetitive light pulses instead of continuous illumination; thus, the required energy is lower. Despite this, Bruegman et al. demonstrated that lower intensity repetitive light pulses had a lower efficiency in terminating AF as compared to higher intensity, conduction block inducing illumination [81].

Another possible solution could be delivering short-lived light pulses in a previously illuminated heart, which would also allow optical mapping and would identify the exact location of the excitable gap. However, achieving full illumination is difficult, especially in the beating heart [81].  Table 3 summarizes the main studies focusing on biological AF cardioversion.

#### 3.2.3. Hyperpolarizing Opsins

**Figure 2:**
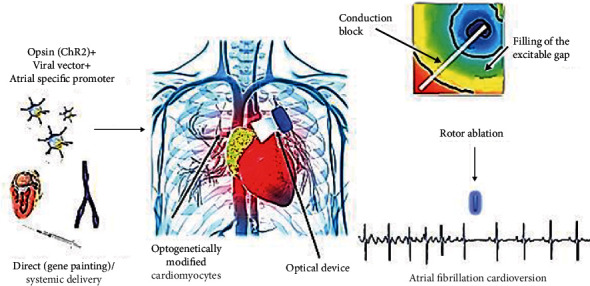
Cardiac optogenetics in atrial fibrillation cardioversion. CHhR2: channelrhodopsin 2.

Attempts have been made to terminate AF using hyperpolarizing opsins, such as channelrodopsin and halorhodopsins. Unlike ChR, they are pumps, which require 1 phonon per each anion, resulting in lower intensity currents. If a proper intensity would be achieved, AF could be terminated by stabilizing membrane resting potential using hyperpolarizing opsins. Moreover, Gruber et al. even proposed that these opsins could be used to mimic pulmonary vein ablation lesions, thus creating a conduction block. However, the same authors highlight the possible proarrhythmic characteristics of hyperpolarizing opsins through the *anode-break excitation* effect [26, 27]. Although the authors have successfully shown that hyperpolarizing opsins could inhibit cardiomyocyte excitation [27, 30], the success rate is low in terminating AF, most likely because of reduced intensity currents.  Figure 2 illustrates the uses of cardiac optogenetics in AF cardioversion.

## 4. Translational Challenges and Side Effects

### 4.1. Translational Challenges

There are several challenges to be addressed before considering using optogenetics in clinical practice, including the choice of opsins, the delivery strategy and the preferred vector, subsequently the possible immune responses to both opsins and vectors, and the difficulties of developing an illumination device that would be feasible in the beating heart. Moreover, it has to be highlighted that most studies have been performed on cultured cardiomyocytes, computational models (in silico), and very few on beating hearts—either mice or rats.

The most widely used opsin is ChR2 with the H134R mutation, a nonspecific depolarizing cation channelrhodopsin [11]. The generated current is much intense than in the case of hyperpolarizing opsins, which are in fact pumps and require 1 photon for the influx of each anion. ChR2 opens upon blue light stimulation, but with weaker penetration. As such, red light-shifted channelrhodopsins may be used, which respond to red light simulation. The authors have used both ChR2 and ReaCh in testing for the success of AF termination. Interestingly, some report in a computational LGR-CMR model that the use of ReaCh might not be necessary and that ChR2 could be used for successful atrial arrhythmia termination. However, in the mentioned study, the authors conducted AT termination by delivering targeted light pulses to the previously identified arrhythmia isthmus [78]. Other opsin variants, including a new variant of opsin allowing calcium influx (CatCh), have also been successfully used. In addition, researchers modified the classical ChR2 in order to prolong the open state upon the same illumination conditions [15].

The key to successful AF optogenetic cardioversions is transmural opsin expression. This can be achieved by ensuring the use and the proper delivery of the preferred opsin, choice of appropriate vectors and promoters, and transmural illumination [88]. Regarding gene delivery, local delivery has some advantages over systemic delivery [30]. Using a *gene painting* technique ensures delivery to the desired area with little to near absent expression in surrounding areas, as shown in this study in which right atrium-gene painting delivery led to 0.01% ChR2 left ventricular expression [34]. Moreover, the required vectors and opsin concentration are lower and the reported immune reactions are of smaller intensities [34, 42, 88–91]. On the other hand, local delivery might lead to inhomogeneous opsin expression in the delivered region (i.e., right atrium), as opposed to systemic delivery, while the latter might lead to inhomogeneous opsin expression between the atria and the ventricles [91]. In the case of systemic AAV delivered opsins, the use of atrial-specific promoters, such as sarcolipin or NPPA, could help limit opsin expression to the atria. Another strategy could be direct delivery of opsin-expressing cells, such as fibroblasts, that by connecting to the neighbouring cardiomyocytes will ensure contraction upon specific wavelength stimulation [92, 93].

### 4.2. Side Effects

Immune reactions can target both delivered opsins and vectors and directly influence the lifespan of appropriate opsin expression [42, 64, 90]. So far, there have been no severe immune reactions reported that could render optogenetics inefficient, but it must be noted that most studies focused either on cellular cultures, including retinal cells, an immune-privileged organ, or involved rats or mice. In other words, the exact immune reactions against opsins and their vectors are still under study in humans. For example, although focusing on immune reactions involving central and peripheral nervous system opsin delivery, Maimon et al. bring into discussion the immune reactions rendering light-based stimulation inefficient and even bring into discussion the necessity of a concomitant immunosuppressive therapy like tacrolimus [94]. The authors showed that tacrolimus-treated rodents had longer opsin expression. Moreover, the authors highlight the possibility of developing antibodies against both AAV and opsins, with different associated risks. For example, developing antibodies against ChR2 may be associated with cellular apoptosis.

The fact that opsin expression depends greatly on the species was demonstrated by several authors. Bruegmann et al. [46, 81] and Vogt et al. [44] revealed long-term expression of ChR2 allowing successful pacing and defibrillation at 15 months in mice, while Nussinovitch et al. reported only eight weeks in rats [27].

Boyle et al. highlighted the CD45+ lymphocyte infiltrates weeks after opsin delivery [86]. In addition, humans might have AAV antibodies from previous infections while rodents showed no antibodies against AAV9 [91]. At the same time, it must be emphasized that ideal candidates for optogenetic cardioversion would already have a proinflammatory state associated with underlying heart failure and AF. It becomes problematic if the antibodies are directed against the already few cardiac-specific serotypes, such as AAV2 or AAV9; it could be efficient in humans to test for the presence of these antibodies before choosing a promoter. Another solution may be synthetically developing new AAV capsules.

There is concern over the phenotoxicity and phototoxicity associated with opsin and light pulse delivery [91]. Opsin overexpression may lead to cellular phenotoxicity through endoplasmic reticulum alterations [92], although the authors point out that cardiomyocyte ChR2 H134 overexpression is not harmful [93]. It must be highlighted though that further studies need to evaluate the effects of other opsin overexpression on cardiomyocytes, such as ReaCh and CatCh [95].

Phototoxicity includes both chemical and thermical injuries [83]. Chemical injuries may occur through the light pulse side stimulation of naturally occurring flavins, nicotine-amide adenine dinucleotide NADP, and melanin. Nearly 50 *μ*M of flavin is found in the heart, which may determine ROS production upon stimulation with 300-500 nm light, the same spectrum as ChR2 [84, 85]. Thermical injuries are possible with light stimulation, and they may also enhance ROS production, while a 10°C increase in temperature and an overall 48°C have been shown to lead to irreversible cellular damage [91]. However, it has been revealed that light stimulation induces only a slight 0.4°C increase with 470 nm, 3.5 mW/mm^2^, 1000 ms light pulses [34]. Providing appropriate illumination in the beating heart might be difficult. It may be achieved through a micro-LED implantable device, but setting the correct wavelength and accounting for interference is recommended. Optical fibre light source devices have been proposed, resembling pacemaker leads. In this case, it may be preferable to insert them epicardially, due to the ability of blood to absorb light. Micro-LED elastic membranes have been designed to envelope the heart, capable of delivering both localized and general illumination [61]. The same authors have designed wireless LED implantable devices.

Aside from the immunological reactions that can render opsins inefficient and may even harm cardiomyocytes, the authors have observed that over time, opsin expression may suffer a process of loss of function, and its connection to the possible immune reactions is to be studied [94]. They bring into discussion several possible other causes of loss of function, including direct cytotoxicity-excitotoxicity, opsin-directed toxicity, epigenetic silencing, episomal DNA loss, and anatomical scattering [94].

At the same time, the same study raised the possible cellular damage induced by illumination-determined cellular acidosis.

Attention has been raised towards the potential proarrhythmic risk of optogenetics [91]. ChR2 is, in fact, a nonspecific cation, which means that upon blue light stimulation it allows cation influx, including Na and Ca [96]. This calcium accumulation might in fact be proarrhythmic; however, no long-terms effects have been observed [89]. In this regard, the authors have reported no ventricular ectopic activity after gene delivery outside of light stimulation and a few atrial ectopic beats when applying light pulses outside the arrhythmic event, but with overall sinus rhythm maintenance [34]. There are several issues that so far remain unaddressed.

If optogenetic AF cardioversion will be used in AF patients, further studies need to be implemented in fibrotic hearts, to account for technical difficulties in achieving successful AF termination in the context of atrial fibrosis. Another issue rests with the long-term expression of opsins. Although rodent studies have shown that opsin expression is stable over time, humans have much longer lifespans and have many other possible technological interferences in our day-to-day lives.

Following the same idea, the used models so far have been either cardiomyocyte monolayers or rodent hearts, which have smaller dimensions and thus require smaller amounts of genes, vectors, promoters, and light intensities. Implementing cardiac optogenetics on bigger hearts, such as swines', is a next foreseeable and required step before introducing cardiac optogenetics in humans. The applicability of these techniques in humans remains to be seen; the fact that human atria are thicker than rodent's mean that most probably higher intensity light pulses might be needed, most likely over a wider atrial surface. However, ventricular arrhythmia termination has been reported in mice, and rodent ventricles have similar thickness to human atria; therefore, the required intensities and safety profiles might be comparable.

## 5. Conclusions

Cardiac optogenetics is the most expected alternative for AF cardioversion, especially in young, highly symptomatic, drug refractory, and heart failure patients. It would allow shock-free, out-of-hospital cardioversion, with the ability of expressing both depolarizing and hyperpolarizing opsins, such ChR2 and Arch, respectively. This would enhance the control of the heart's electrical activity. Researchers have already attempted a hybrid model capable of both detecting and terminating AF based on cardiac illumination, highlighting that the three most important parameters to consider for a successful biologic defibrillation are light intensity, light pulse duration, and the applied surface. It is agreed upon that to achieve successful cardioversion the duration of the applied light pulse must be longer than the arrhythmic cycle. Although it seems that both immune reactions and proarrhythmic effects are minimal, more studies are required to determine, first, the safety profile of cardiac optogenetics in humans and, second, the exact parameters of light stimulation that would allow high-accuracy AF cardioversion.

## Figures and Tables

**Table 1:** Types of opsin actuators used in cardiac optogenetics.

Type of opsin		Class	Proteins	Effect on membrane potential
Pump	Proton	Bacteriorhodopsins	Arch, ArchT	Hyperpolarizing
	Chloride	Halorhodopsins	eNpHR3.0	
Channel		Channelrhodopsins	Ex. Channelrhodopsin 2 (ChR2)	Depolarizing

**Table 2:** Comparison between systemic and direct opsin delivery.

Systemic delivery		Direct intramyocardial delivery	
+	−	+	−
Uniform	(i) To be specific, it requires a vector with cardiac tropism and/or promoters (ii) Requires high concentrations to achieve uniform expression due to intravascular dilution (iii) Extracardiac expression (iv) Possible immune reaction (v) Cannot be used to achieve local myocardial expression	(i) Lower viral dose required (ii) Increased transduction (iii) Lower incidence of immune response	(i) Inhomogeneous distribution (inefficient if cardiac defibrillation is desired)

**Table 3:** Studies researching optogenetic AF cardioversion.

Author, year	Opsin	Light pulse characteristics	Additional remarks	Reference
Nyns et al., 2019	ReaChR2	470 nm, 2.5 mW/mm^2^, 20 mm^2^, 1000 ms	AF termination success rate dropped with the decrease in the surface	[35]
Boyle et al., 2018	ChR2	488 nm, 1.5 mW/mm^2^, 1000 ms	LGE-CMR fibrotic heart atria tachycardia computational model	[86]
Bruegman et al., 2018	ChR2	470 nm, 0.4 mW/mm^2^, 1000 ms, 100 mm^2^	>0.4 mW/mm^2^ light pulses were the most successful in AF termination Authors used epicardial illumination Reducing light pulse time reduced the cardioversion success rate	[81]
Houston et al., 2018	ChR2	460 nm, 0.42 mW/mm^2^ up to 0.79 mW/mm^2^, 274 mm^2^, 500 ms	0.79 mW/mm^2^ light pulses had the highest success rate ChR2 is most active seconds after activation	[84]
Feola et al., 2017	CatCh	470 nm, 0.3 mW/mm^2^, 3, 6, 12 mm, 500 ms	Conduction line block including the rotor core and at least one unexcitable edge	[83]
Bruegman et al., 2016	ChR2	460 nm, 0.40 mW/mm^2^, 143 mm^2^	—	[46]
Bingen et al., 2014	CatCh	470 nm, 38 *μ*W/mm^2^, 500 ms	Successful AF termination using very low intensity blue light pulses in rodent atrial cardiomyocytes	[87]

## Data Availability

The literature data supporting this review are from previously reported articles, which have been cited.
